# Reducing the Cost of Proton Radiation Therapy: The Feasibility of a Streamlined Treatment Technique for Prostate Cancer

**DOI:** 10.3390/cancers7020688

**Published:** 2015-04-24

**Authors:** Wayne D. Newhauser, Rui Zhang, Timothy G. Jones, Annelise Giebeler, Phillip J. Taddei, Robert D. Stewart, Andrew Lee, Oleg Vassiliev

**Affiliations:** 1 Department of Physics and Astronomy, Louisiana State University, 202 Nicholson Hall, Baton Rouge, LA 70803, USA; E-Mails: rzhang@marybird.com (R.Z.); ovassiliev@marybird.com (O.V.); 2 Department of Physics, Mary Bird Perkins Cancer Center, 4950 Essen Lane, Baton Rouge, LA 70809, USA; 3 Departments of Radiation Physics and Radiation Oncology, The University of Texas MD Anderson Cancer Center, 1515 Holcombe Blvd, Houston, TX 77030, USA; E-Mails: bandnerdtmi@gmail.com (T.G.J.); Giebeler.Annelise@scrippshealth.org (A.G.); pt06@aub.edu.lb (P.J.T.); aklee@mdanderson.org (A.L.); 4 The University of Texas Graduate School of Biomedical Sciences, Houston, TX 77030, USA; 5 Department of Physics, Abilene Christian University, ACU Box 27963, Abilene, TX 79699, USA; 6 Department of Radiation Oncology, University of Washington School of Medicine, 1959 NE Pacific Street, Box 356043, Seattle, WA 98195, USA; E-Mail: trawets@uw.edu

**Keywords:** proton therapy, prostate cancer, multileaf collimator, cost

## Abstract

Proton radiation therapy is an effective modality for cancer treatments, but the cost of proton therapy is much higher compared to conventional radiotherapy and this presents a formidable barrier to most clinical practices that wish to offer proton therapy. Little attention in literature has been paid to the costs associated with collimators, range compensators and hypofractionation. The objective of this study was to evaluate the feasibility of cost-saving modifications to the present standard of care for proton treatments for prostate cancer. In particular, we quantified the dosimetric impact of a treatment technique in which custom fabricated collimators were replaced with a multileaf collimator (MLC) and the custom range compensators (RC) were eliminated. The dosimetric impacts of these modifications were assessed for 10 patients with a commercial treatment planning system (TPS) and confirmed with corresponding Monte Carlo simulations. We assessed the impact on lifetime risks of radiogenic second cancers using detailed dose reconstructions and predictive dose-risk models based on epidemiologic data. We also performed illustrative calculations, using an isoeffect model, to examine the potential for hypofractionation. Specifically, we bracketed plausible intervals of proton fraction size and total treatment dose that were equivalent to a conventional photon treatment of 79.2 Gy in 44 fractions. Our results revealed that eliminating the RC and using an MLC had negligible effect on predicted dose distributions and second cancer risks. Even modest hypofractionation strategies can yield substantial cost savings. Together, our results suggest that it is feasible to modify the standard of care to increase treatment efficiency, reduce treatment costs to patients and insurers, while preserving high treatment quality.

## 1. Introduction

Proton radiation therapy is a safe and effective treatment for cancers of the central nervous system [1], eye [2], prostate [3], and other anatomical sites [4,5,6,7,8]. The availability of proton therapy is limited to 16 centers in the United States and 46 centers worldwide (www.ptcog.ch). Most aspects of proton therapy are well understood, including the physics clinical operation, accelerator and beamline engineering, treatment planning, and radiation protection of staff and patients [9,10,11,12,13,14]. Several medical manufacturers offer proton therapy systems at costs ranging from approximately $20M to $100M (US), depending on the capacity and capability of the equipment. These costs are much higher than those of conventional radiotherapy equipment based on electron linacs, which presents a formidable barrier to most clinical practices that wish to offer proton therapy.

In the long term, economies of scale may help to drive down the cost of proton therapy installations. In the near term, and for existing facilities, cost reductions can be achieved by decreasing the required time, staff effort, and materials required for a course of proton therapy. However, the bulk of an extensive body of literature documents methods to improve the quality of proton dose distributions, e.g., using sophisticated scanned beam delivery techniques [8,15,16], while less attention has been paid to analyses of cost, e.g., comparisons of proton and photon radiotherapies [17,18,19,20]. Still less has been reported on the role of multileaf collimators (MLC) [21,22,23,24,25,26] and range compensators in the cost of a course of proton therapy. Prostate cancer is of particular interest in this regard because of its high incidence and the large proportion of proton treatment capacity utilized for its treatment.

Another possible way to increase the efficiency and cost effectiveness of fractionated radiotherapy is to develop hypofractionated treatments as the standard of care (SOC) [27]. Substantial evidence points towards a low α/β for prostate cancer [28], and a number of clinical trials provide encouraging evidence for the use of moderate (2.5 to 3.16 Gy/fx) to extreme (7 to 9 Gy/fx) hypofractionation with photon-based treatments [28,29]. Proton therapy produces dose distributions that are as good as or better than most photon-based treatments, and it is reasonable to expect the hypofractionation strategies from photon therapy are applicable to proton therapy.

The objective of this study was to evaluate the feasibility of cost-saving modifications to the present SOC for proton treatments for prostate cancer. In particular, we quantified the dosimetric impact of a streamlined treatment technique in which custom-fabricated collimators were replaced with a MLC and the custom-fabricated range compensators were eliminated. The dose distributions were calculated with a clinical treatment planning system (TPS) and confirmed with corresponding Monte Carlo simulations. The lifetime risks of radiogenic second cancer were calculated based on dosimetric results and existing dose-risk models based on epidemiologic data. To guide the development of hypofractionated proton therapy treatments, estimates of the range of proton fraction sizes and total treatment doses equivalent to a conventional photon treatment of 79.2 Gy in 44 fractions (1.8 Gy/fx) were determined using isoeffect methods.

## 2. Methods and Materials

### 2.1. Patient Cohort

The patient cohort included 10 patients with low-risk prostate cancer who were treated at The University of Texas MD Anderson Cancer Center (MDACC). Patients for the cohort were selected using the consecutive sampling method. The inclusion criteria were a diagnosis of localized adenocarcinoma of the prostate, treatment with passively scattered proton beam therapy (PSPT), and under the direction of one single radiation oncologist to avoid intra-physician variations in contouring of the clinical target volume (CTV) and other structures. Rejection criteria included patients with hip prostheses, which can lead image artifacts and dose errors [30]. Data for this retrospective study was collected according to a protocol approved by the MD Anderson Institutional Review Board and anonymized using standard methods [31] for the study.

### 2.2. Treatment Planning

All treatment plans in this work were prepared using a commercial TPS (Eclipse Version 8.9, Varian Medical Systems, Palo Alto, CA, USA) that was previously commissioned for clinical use [32]. The TPS included a pencil beam algorithm [9] to calculate the dose distribution in the patient. In general, the system has excellent accuracy when compared with measurements. In addition, the accuracy in complex soft tissue is excellent when compared with Monte Carlo simulations. The accuracy in regions with large density heterogeneities is probably an area where further study might be beneficial. However, for the anatomy considered in this study, the Eclipse pencil beam algorithm is more than sufficiently accurate for calculating the therapeutic dose. Each patient’s treatment plan utilized the lateral opposed-pair PSPT field arrangement. The prescription was 76 Gy (RBE) mean absorbed dose to the CTV, which included the prostate and proximal seminal vesicles, to be delivered in 2 Gy (RBE) fractions. All plans were reviewed and approved by a board-certified radiation oncologist (AKL). The proton beam penetration ranges varied from 22.0 cm to 27.1 cm water equivalent thickness (WET), the SOBP widths varied from 8 cm to 10 cm WET, and CTV volumes varied from 46.3 cm^3^ to 81.8 cm^3^. For each patient, each of the following three separate treatment techniques were considered.

The first technique was the current SOC at MD Anderson Cancer Center, namely, beam collimation with a custom brass aperture block and distal-edge field shaping with a Lucite range compensator (RC).

**Figure 1:**
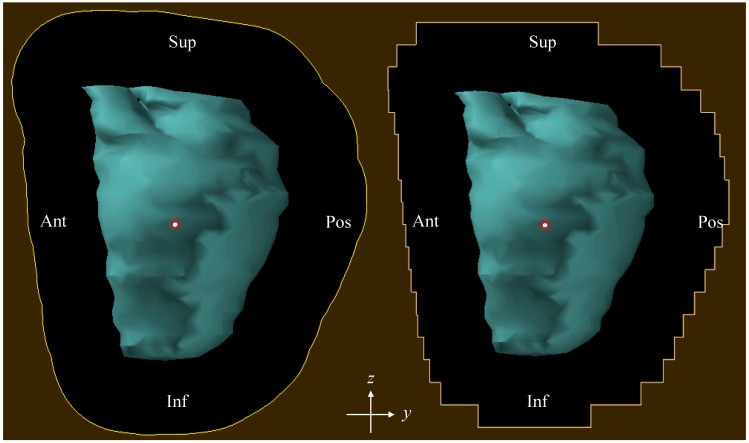
The custom brass aperture used in SOC treatment plan (left) and the MLC leaf positions used in “MCL+RC” and “MLC only” treatment plans (right). The CTV is shown in light blue. (Sup: superior; Inf: inferior; Ant: anterior; Pos: Posterior).

The second technique was as before except the custom brass aperture was replaced with a static multileaf collimator (MLC + RC). The MLC was composed of brass, leaf widths were 0.5 cm, and the leaf positions were chosen so that the midpoint of each edge intersected the aperture line from the SOC plan (Figure 1). For one patient, we evaluated the impact of the orientation of the leaves with respect to the patient (along the *y* direction (anterior-posterior) or along the *z* direction (inferior-superior)). We found that the orientation’s impact on the dose distributions was negligible. Therefore, we used only one orientation (along the *y* direction) for all patients.

The third technique also utilized the MLC but eliminated the use of range compensators (MLC-only). The MLC-only plans were created by manually editing the range compensators to have uniform thickness of 1.0 mm, which closely approximated the complete absence of a range compensator, yet satisfied the Monte Carlo system’s requirement to have a range compensator present for each field. The treatment nozzle’s position was the same for MLC + RC and MLC-only plans. Other details of the treatment planning technique were described elsewhere [33].

### 2.3. Monte Carlo Simulations

Monte Carlo simulations were performed on one patient for the SOC and MLC-only plans to confirm the accuracy of the pencil beam dose algorithm to predict the therapeutic distribution of therapeutic dose. In addition, corresponding Monte Carlo simulations were performed for both plans to estimate the stray and leakage neutron radiation exposures. These exposures, which are not calculated by the TPS, were needed to quantify differences in the predicted risk of radiogenic second cancer when using an MLC *versus* a static collimator. The neutron dose from MLC + RC plan was expected to be very similar to SOC plan and was therefore not simulated. All simulations in the work were performed with the Monte Carlo Proton Radiotherapy Treatment Planning (MCPRTP) system [34], which used the Monte Carlo N-Particle eXtended (MCNPX) code version 2.6b [35] with parallel processing as a radiation dose calculation engine. More details of Monte Carlo simulations can be found in previous reports from our group [36,37].

In separate Monte Carlo simulations for each field of each plan, we calculated absorbed dose from therapeutic protons, equivalent dose from neutrons created in the treatment unit (“external neutrons”), and equivalent dose from neutrons created in the phantom (“internal neutrons”). The simulated doses were normalized so that each field delivered a mean therapeutic proton absorbed dose of 34.5 Gy in the CTV (*i.e*., a total of 69 Gy in the CTV for both fields of each plan). The equivalent dose from neutrons was weighted by a mean radiation weighting factor for neutrons of 6.2 taken from the literature [38]. This weighting factor represents an average value from external and internal neutrons and is therefore appropriate for the purposes of this study. Dose distributions calculated by the MCPRTP system were imported into the TPS, and mean doses were calculated in the CTV, bladder, and rectum for each radiation type and each plan.

### 2.4. Calculations of Lifetime Risk of Neutron-Induced Second Cancer of the Bladder and Rectum

The predicted lifetime risk of radiogenic second cancer incidence in the bladder and rectum from neutrons was calculated as product of the mean organ equivalent dose and the organ-specific risk coefficient for lifetime attributable risk from the Biological Effects of Ionizing Radiation-VII Report (BEIR VII) [39]. These risk coefficients were applied with the following exception. Because the risk coefficient used for rectal cancer in the BEIR VII Report was intended to be applied to the dose in the entire colon, applying it directly to only the dose in the rectum, which is nearer to the field than the rest of the colon, would result in a slight overestimation of risk. Instead, we applied a fractional mass correction factor of 0.2 (*i.e*., accounting for the rectum being 20% of the mass of the entire colon, including the rectum) to calculate risk for the rectum [40,41]. Specifically, for a 60-year-old man they were 0.19%/Sv and 0.66%/Sv for lifetime attributable risk for developing cancers of the rectum and bladder, respectively.

### 2.5. Determination of Equivalent Photon and Proton Prescription Doses

For the special case of a slowly dividing tumor cell population, the total photon dose *D*_γ_ delivered in *n* fractions that is expected to produce the same level of tumor control (*i.e*., same proportion of reproductive cell death) was estimated using an isoeffect model [42]:

(1) $$D_{\gamma }(n)=\frac{n}{2}{\left(\alpha /\beta \right)}_{\gamma }\left\{-1+\sqrt{1+\frac{4BED_{\gamma }}{n{\left(\alpha /\beta \right)}_{\gamma }}}\right\},$$

where (α/β)_γ_ is the linear-quadratic (LQ) model parameter that determines the sensitivity of a cell or tissue to fraction size, and *BED*_γ_ ≡ *D_R_*[1 + *d_R_*/(α/β)_γ_] is the biologically equivalent photon dose for a clinically acceptable prostate cancer treatment (total absorbed dose *D_R_* delivered in fractions of size *d_R_*). Once an equivalent photon dose for *n* fractions is computed using Equation (1), the equivalent proton absorbed dose, *D_p_*(*n*), can be computed by multiplying *D*_γ_ by the proton relative biological effectiveness (RBE), *i.e.*, *D_p_*(*n*) = RBE(*n*) × *D*_γ_(*n*). All of the results in this work are based on the recommended generic clinical RBE of 1.1 [10], which is currently used in the standards of care for proton therapy at many institutions, including MDACC.

## 3. Results

### 3.1. Comparison of SOC Treatment Plans to MLC + RC and MLC-only Treatment Plans

**Figure 2:**
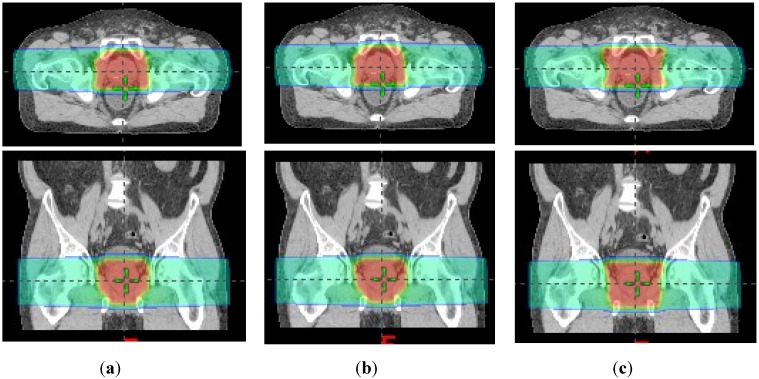
Dose distributions on axial (upper) and coronal (lower) slices from (**a**) standard of care; (**b**) MLC + RC and (**c**) MLC only treatment plans.

**Figure 3:**
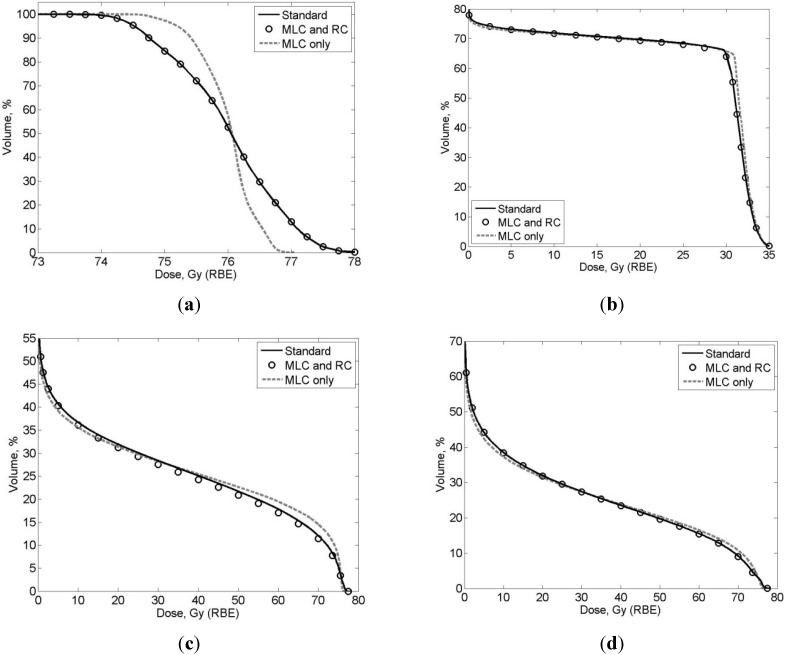
Dose volume histogram (DVH) comparison of (**a**) CTV; (**b**) femoral heads; (**c**) bladder and (**d**) rectum between different treatment techniques. The DVHs here were from the patient with the largest discrepancy between techniques.

The SOC, MLC + RC, and MLC-only treatment plans were all clinically acceptable. Figure 2 shows the dose distributions from all three techniques on axial and coronal slices. The SOC and MLC + RC plans have almost identical dose distributions, while MLC only plan delivered more dose to the muscle lateral to the PTV. Table 1 lists the therapeutic organ and tissue doses, revealing a high degree of similarity between the plans. Figure 3 plots DVHs that further evidence the dosimetric similarities. Because of these strong similarities in therapeutic dose distributions, the risks of second cancer incidence from therapeutic doses were not predicted; only risks from neutron equivalent doses were estimated (Section 2.4).

**Table 1:** Mean dose values (average over all 10 patients) for three treatment techniques: standard of care (SOC) with custom aperture and range compensator, MLC with range compensator (MLC + RC), and MLC without range compensator (MLC only). All plans were normalized such that the mean dose to the clinical target volume was 76 Gy (RBE).

Organ	SOC		MLC + RC		MLC Only	
	Max	Mean	Max	Mean	Max	Mean
Anterior Rectal Wall	76.77	39.48	76.89	75.90	76.89	75.90
Bladder	77.09	12.87	76.35	12.18	76.35	12.06
CTV	77.63	75.90	76.89	75.90	76.89	75.90
Distal Seminal Vesicles	75.37	67.63	75.87	68.74	75.80	68.29
Femoral Heads	36.94	25.28	42.16	25.38	42.15	25.30
Proximal Seminal Vesicles	76.92	75.83	76.46	75.85	76.45	75.83
Rectum	76.81	16.73	76.34	16.81	76.33	16.76

**Figure 4:**
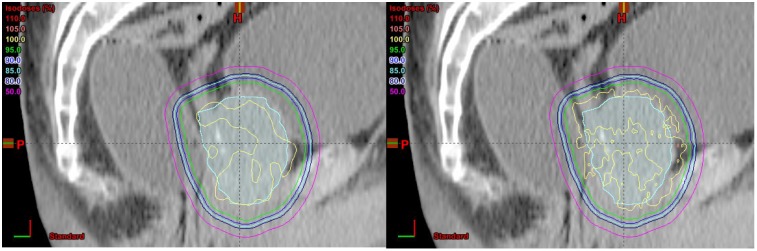
Dose distributions calculated by pencil beam algorithm (left) and Monte Carlo simulation (right) on a sagittal slice.

Figure 4 shows dose distributions calculated by pencil beam algorithm (left) and Monte Carlo simulation (right) on a sagittal slice and we can see that there is no scalloping effect for the beam energy and setup used in this study. The dose distributions are very similar between pencil beam algorithm and Monte Carlo simulation.

Table 2 lists the Monte Carlo predictions of the mean stray neutron equivalent doses from the SOC plan and the MLC-only plan. Values are listed separately for each plan and for internal and external neutrons. The statistical uncertainties of the equivalent dose in the voxel at the treatment isocenter were 0.46% or less for the proton simulations and between 8% and 12% for the neutron simulations (one standard deviation), resulting in a low statistical uncertainty in the mean neutron equivalent dose in the contoured structures, which comprised many voxels. In all the organs/tissues, the MLC-only plan decreased the external neutron equivalent dose and increased the internal neutron equivalent dose compared to the corresponding SOC plan. However, at less than 52 mSv difference, the predicted lifetime risks of rectal or bladder cancer incidence from neutrons were virtually identical at 0.51% for both the SOC plan and the MLC-only plan. These predicted absolute risk values are consistent with what may be deduced roughly from previous outcomes-modeling and epidemiology studies [33,43,44].

**Table 2:** Mean equivalent dose from stray neutrons in mSv for one prostate patient.

Organ/Tissue	SOC			MLC Only		
	External	Internal	Total	External	Internal	Total
Anterior Rectal Wall	228	561	788	174	641	815
Bladder	222	363	585	170	413	582
CTV	228	840	1067	172	938	1110
Distal Seminal Vesicles	233	691	924	173	787	960
Femoral Heads	280	531	811	217	572	789
Prostate	227	841	1068	173	941	1113
Proximal Seminal Vesicles	239	814	1053	169	873	1042
Rectum	223	425	648	171	481	652

### 3.2. RBE Effects in Conventional and Hypofractionated Proton Treatments

**Figure 5:**
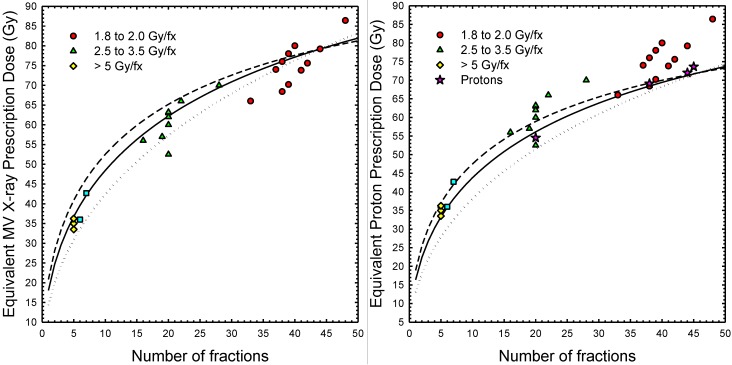
Photon dose (*left panel*) and proton dose (*right panel*) for prostate cancer equivalent to a photon treatment of 79.2 Gy delivered in 44 fractions (1.8 Gy/fx). Filled circles, triangles, squares and diamonds (*both panels*) denote treatment protocols from past standard of care (SOC) and ongoing clinical trials with MV X-rays [28]. Filled stars (*right panel only*) denote SOC treatments or treatments under consideration for clinical trials at selected proton centers (MPRI, MD Anderson, Loma Linda). **Left panel:** dotted line (α/β)_γ_ = 1 Gy; solid line (α/β)_γ_ = 3 Gy; dashed line, (α/β)_γ_ = 5 Gy. Doses above solid black lines more likely to achieve local control, and doses below the solid black lines are less likely to achieve local control. Dashed and dotted lines indicate uncertainties associated with tumor α/β in the range from 1 to 5 Gy [28]; **Right panel:** dotted line (α/β) = 1 Gy; solid line (α/β) = 3 Gy; dashed line, (α/β) = 5 Gy. Multiply the proton doses in the *right panel* by ~1.1 for photon equivalent dose (Gy (RBE)).

The extrapolated interval of equivalent photon doses determined using Equation (1) with 1 Gy ≤ (α/β)_γ_ ≤ 5 Gy are in good agreement with the doses selected for many clinical trials (Figure 5, left panel). From Table 3, a SOC photon treatment of 79.2 Gy in 44 fractions is equivalent to 66.9 Gy to 69.2 Gy in 38 fractions, depending on the α/β value used. Alternatively, the SOC for proton therapy at MD Anderson calls for 76 Gy (RBE = 1.1) in 38 fractions, which from Equation (1) is approximately equivalent to a photon absorbed dose of 68.1 Gy to 68.6 Gy in 38 fractions (α/β = 15 Gy). As illustrated in Figure 5, the isoeffect model predicted strong similarities between photon and proton hypofractionation regimens. For example, a hypofractionated proton therapy regimen comprising 20 fractions at 2.6 Gy to 2.9 Gy of absorbed dose per fraction (2.86 Gy (RBE)/fx to 3.19 Gy (RBE)/fx) is comparable to the corresponding hypofractionated photon therapy regimen.

**Table 3:** Proton therapy prescriptions doses for prostate cancer equivalent to a 79.2 Gy in 44 fractions (1.8 Gy/fx) photon treatment. Multiply the tabulated proton dose by ~1.1 for photon equivalent dose (Gy RBE). Minimum, middle and maximum dose estimates correspond to a photon α/β = 1 Gy, 3 Gy and 5 Gy, respectively [28]. Ratio of maximum to minimum treatment dose is a measure of the prescription uncertainty associated with the estimates α/β for the prostate; values of this ratio greater than about 1.05–1.1 may be considered clinically significant (comparable in magnitude to dose delivery errors).

*n*	Fraction Size (Gy)			Total Dose (Gy)			Ratio
	min	mid	max	min	mid	max	max/min
1	13.0	16.4	18.9	13.0	16.4	18.9	1.45
3	7.3	8.9	10.1	22.0	26.8	30.3	1.38
5	5.6	6.7	7.4	27.9	33.3	37.0	1.33
10	3.8	4.4	4.8	38.2	43.8	47.5	1.25
15	3.0	3.4	3.6	45.6	50.8	54.2	1.19
20	2.6	2.8	2.9	51.5	56.1	58.9	1.14
25	2.3	2.4	2.5	56.5	60.4	62.6	1.11
30	2.0	2.1	2.2	60.9	63.9	65.5	1.08
35	1.9	1.9	1.9	64.8	66.8	68.0	1.05
38	1.8	1.8	1.8	66.9	68.4	69.2	1.03
40	1.7	1.7	1.8	68.3	69.4	70.0	1.03
44	1.6	1.6	1.6	70.8	71.2	71.4	1.01
45	1.6	1.6	1.6	71.5	71.7	71.8	1.00

A recent meta-analysis of *in vitro* and *in vivo* data on proton RBE [45] found some support for dose-averaged RBE values that ranged from about 1.1 at the proximal edge of a typical SOBP to 1.15 at the center of the SOBP (~2 Gy per fraction). Towards the distal edge of the SOBP, laboratory studies of cell survival suggest that proton RBE increases towards 1.35 towards the distal edge of the SOBP and may reach a value as high as 1.7 in the fall-off region of the SOBP [45]. When the fraction size is less than α/β, proton RBE tends to increase with decreasing alpha/beta. When the fraction size becomes comparable to or larger than alpha/beta, proton RBE is relatively insensitive to fraction size [45]. For a hypofractionated prostate cancer (α/β~1–5 Gy) treatment that delivers 52–58 Gy in 20 fractions (<2.6–2.9 Gy/fx), an assumed SOC RBE of 1.1 is not inconsistent with SOBP estimates based on *in vitro* cell survival. For extreme hypofractionation, the majority of the laboratory studies indicate that the tumor RBE gradually approaches an asymptotic value in the range from 1.0 to 1.1 as the fraction size becomes large compared to α/β [45]. When viewed against the other inter-institutional practices (e.g., Figure 5 left panel) and sources of dosimetric uncertainty, a SOC RBE of 1.1 seems quite reasonable–at least until models and treatment planning tools that explicitly account for spatial variations in proton RBE become available and are validated in studies of clinical outcomes.

## 4. Discussion

In this study, we compared three different proton therapy treatment plan techniques for prostate cancer, namely the current SOC, MLC + RC and MLC-only techniques. We also performed isoeffect calculations to compare SOC and hypofractionated treatment regimens for photon and proton therapies.

The major findings of this study showed that the predicted dosimetric impacts (on therapeutic and stray doses) of removing the RC and replacing the custom collimator with an MLC are clinically insignificant. These findings are important because they strongly suggest that it is possible to reduce total treatment times by eliminating the need to change custom milled collimators and RCs before and/or during daily irradiations. Additional cost savings may be expected from reduced use of raw materials and milling equipment and the associated building spaces they occupy.

Another major finding was that isoeffect calculations suggest that past and ongoing photon trials with moderate to extreme hypofractionation provide a strong rationale for considering similar hypofractionated treatments using proton therapy. Such hypofractionation schemes can potentially reduce the cost of proton radiotherapy. For example, for each eliminated fraction, a patient traveling from out of town to a proton therapy center may save $51 to $71 per day on meals and incidentals, $80 to $185 per day on lodging, and $200 or more per day on lost wages (range of estimate based on US government *per diem* (http://www.gsa.gov) rates for 7 representative cites near proton therapy facilities). A hypofractionated treatment delivered in 25 fractions instead of the SOC 44 fractions has the potential to reduce unreimbursed patient costs by as much as $1900 for meals and incidentals and $4970 for lodging (estimates based on approximate number of days for treatment delivery (including weekends) with 5 days for CT simulation and treatment planning). Savings related to lost wages may be as high as $5400 for a family with a median household income of $52,100 per year. Treatment facilities that adopt hypofractionated treatments as the SOC may see a decrease in some (per fraction) billable services (a savings to health insurers). However, hypofractionation would free up capacity to treat additional patients, perhaps enabling an increase overall patient volumes and revenues of by a factor of approximately 2, depending on changes in fractionation and case mix. Increasing overall throughput is possible for centers with excess treatment demand. For centers with operating at or below their capacity, adopting faster prostate treatment methods could free up capacity for other SOC treatments (e.g., craniospinal irradiation) and research and development. The latter may include much needed clinical trials (e.g., comparative effective studies including proton *versus* photon radio therapies), new treatment techniques for other high-impact malignancies (e.g., the treatments of breast cancer and metastases), and the treatment of benign diseases (e.g., neurosurgical ablation of seizure foci, age-related macular degeneration).

Our results yielded several additional findings of secondary importance. The “scalloped” dose distributions caused by the square shape of the MLC leaf ends were predicted by the treatment planning system’s pencil beam dose algorithm and Monte Carlo dose simulations. A comparison of these revealed dose distributions that were indistinguishable from one another for the intents and purposes of this study. We also found that protons scattered from the edge of the collimator played only a small role near the patient midline, thus confirming an earlier finding in a water phantom from Titt *et al.* [46]. Because of the removal of the RC, the air gap in MLC-only plan was larger than that in SOC and MLC + RC plans, but this had negligible effect on the dosimetric results (the collimators were the same distance from the patient in all plans). In fact, the MLC-only plan provided a slight improvement in coverage of the CTV. Using an MLC and removing the RCs did not significantly increase the risk of second cancer induced by stray neutrons compared to the SOC plan. This provides additional evidence that supports the feasibility of time and cost-saving improvement to proton therapy for prostate cancer.

A limitation of our study is that we considered passively scattered proton therapy and not scanned beam proton therapy. Interest in scanned-beam proton therapy is increasing and some facilities are equipped with treatment heads capable of delivering passive and scanned beams or only scanned beams. However, this is not a serious limitation because most proton therapy treatments of prostate cancer have been and are still delivered with passively scattered beams. In addition, beam scanning and other emerging technologies, such as the proton arc treatments [40,47,48,49,50,51] and algorithmically optimized outcomes [52,53] may substantially alter the requirements on lateral penumbral widths and thereby influence the design of beam delivery systems, including collimators.

The utilization of scanned beam treatments for prostate cancer is increasing, yet continued expansion has met with controversy, mainly regarding costs. Studies that directly compare scanned and scattered proton therapies are scarce and equivocal. For example, Pugh *et al.* [54] recently reported a preliminary subset analysis of a prospective clinical trial; they reported no statistically significant differences between passively-scattered and scanned-beam proton therapies in terms of toxicity or quality of life (both conferred low rates of grade 2 or higher GI or GU toxicity), although the authors suggested that future comparative analyses were warranted in a larger cohort. To fully exploit the theoretical advantages of scanned beams, e.g., as evidenced by superior outcomes, it may be necessary to collimate scanned beams to reduce their lateral penumbral width. The reason for this is that, in some cases and particularly at low proton beam energies, (uncollimated) scanned-beam penumbral widths are inferior to those currently achievable with (collimated) passively-scattered beams [55,56]. The beam penumbra will also be high at high proton beam energies [57]. In the case of prostate treatments, high energy is required and the lateral penumbral widths are among the largest of any common proton beam treatments, and penumbral width and margin size govern the doses to the bladder and rectum [58].

An MLC solution, along the lines of what we described here, may provide a straightforward technological migration path to implement a variety of scanned-beam treatment techniques. Furthermore, an MLC may provide a faster and more flexible alternative to a dedicated proton beam trimmer system [59]. An additional benefit of collimators is that they reduce the out-of-field doses, which are largest at high proton beam energies [44,60].

Given the similar outcomes from contemporary passively-scattered and scanned beam treatments for prostate cancer, and the rapid technological progress being made on both these treatment modalities, it is unclear if or when one will be proven superior to the other, e.g., in terms of patient outcomes (such as tumor control, acute and late toxicities, overall survival), safety, convenience, or cost effectiveness. In contrast, it is clear that the question of superiority cannot be resolved without the benefits of additional technological research, e.g., to reduce the doses to the rectum, bladder, and other healthy tissues. Our results agree well with those from the literature. The scalloped effect of MLC is small, which is consistent with previously studies: Bues *et al.* [21] found the scalloped effect of proton MLC was acceptable and comparable to the photon MLC based on Monte Carlo simulation; Kirk *et al.* [61] reported that proton dose profiles using a tungsten MLC have no worse edge-scattering effects than a non-divergent brass aperture. Diffenderfer *et al.* [22] reported that the measured stray neutron dose generated from a closed tungsten alloy MLC is similar to that generated from a closed brass block. Although the situation in our study was different (open field and brass MLC), the qualitative finding agrees well with Diffenderfer *et al.* in that secondary neutron doses do not increase by using an MLC-only technique compared to an SOC technique with custom fabricated collimators. Our results are generally consistent with those reported by Daarts *et al.* [25]. In that study, based on measurements, 1-D and 2-D dose distributions shaped by an MLC and by brass aperture were compared. Similarly to our study, they concluded that dose distribution shaped by an MLC and by brass aperture were equivalent. The risk values in this study are lower than other predictions in the literature [44] for risk of second cancer from stray radiation after proton therapy for prostate cancer. The reason for this is that our risk estimate includes only risks of cancer of the rectum and bladder (the organs at highest risk) and second cancers in other organs and tissues were not considered.

To our knowledge, this is the first study to show the treatment quality can be maintained after implementing an MLC and removing the RC for proton-beam prostate cancer treatments. Our study was further strengthened by testing this result for a clinically representative sample of patients, and performing patient-specific dose calculations. Both therapeutic and stray radiation exposures were included in the dosimetric evaluations and second cancer risks were evaluated.

## 5. Conclusions

In summary, we demonstrated that, for a sample set of proton-beam prostate cancer treatments, removing the RC or changing to an MLC will not affect the dose distributions or the expected treatment outcome in a clinically significant way. We also calculated a plausible range of proton fraction sizes and total treatment doses equivalent to a conventional photon treatment. While our work focused on prostate cancer treatment, the approaches utilized here can potentially be adapted to other cancer sites.
